# Climate and health: a path to strategic co-financing?

**DOI:** 10.1093/heapol/czae044

**Published:** 2024-11-18

**Authors:** Josephine Borghi, Soledad Cuevas, Blanca Anton, Domenico Iaia, Giulia Gasparri, Mark A Hanson, Agnès Soucat, Flavia Bustreo, Etienne V Langlois

**Affiliations:** Global Health and Development, London School of Hygiene and Tropical Medicine, London WC1H9SH, UK; Social Cohesion, Health and Wellbeing group, International Institute for Applied Systems Analysis, Laxenburg 2361, Austria; INSTITUTO DE ECONOMÍA, GEOGRAFÍA Y DEMOGRAFÍA (IEGD), Consejo Superior de Investigaciones Científicas (CSIC), Madrid 28037, Spain; Global Health and Development, London School of Hygiene and Tropical Medicine, London WC1H9SH, UK; Partnership for Maternal, Newborn, Child and Adolescent Health (PMNCH), WHO, Geneva 1211, Switzerland; Partnership for Maternal, Newborn, Child and Adolescent Health (PMNCH), WHO, Geneva 1211, Switzerland; Partnership for Maternal, Newborn, Child and Adolescent Health (PMNCH), WHO, Geneva 1211, Switzerland; Health and Social Protection, Agence Française de Développement (AfD), Paris 75598, France; Fondation Botnar, Basel 4052, Switzerland; Partnership for Maternal, Newborn, Child and Adolescent Health (PMNCH), WHO, Geneva 1211, Switzerland

**Keywords:** Health financing, climate finance, co-financing, mitigation, adaptation

## Abstract

Leveraging the co-benefits of investments in health and climate can be best achieved by moving away from isolated financing approaches and adopting co-financing strategies, which aim to improve the outcomes of both sectors. We propose a framework for studying co-financing for health and climate that considers the degree of integration between sector funding, and whether arrangements are ‘passive’, when cross-sectoral goals are indirectly affected, or ‘strategic’, when they are pre-emptively supported to build resilience and sustainability. We conducted a rigorous, evidence-focused review to describe co-financing mechanisms according to a framework, including the context in which they have been employed, and to identify enablers and barriers to implementation. We searched the international literature using Pubmed and Web of Science from 2013 to 2023, the websites of key health and climate agencies for grey literature and consulted with stakeholders. Our review underscores the significant impact of climate change and related hazards on government, health insurance and household health-related costs. Current evidence primarily addresses passive co-financing, reflecting the financial consequences of inaction. Strategic co-financing is under explored, as are integrative co-financing models demanding cross-sectoral coordination. Current instances of strategic co-financing lack sufficient funding to demonstrate their effectiveness. Climate finance, an under used resource for health, holds potential to generate additional revenue for health. Realizing these advantages necessitates co-benefit monitoring to align health, climate mitigation and adaptation goals, alongside stronger advocacy for the economic and environmental benefits of health investments. Strategic co-financing arrangements are vital at all system levels, demanding increased cross-sectoral collaboration, additional funding and skills for climate integration within health sector plans and budgets, and mainstreaming health into climate adaptation and mitigation plans. Supporting persistent health needs post-disasters, promoting adaptive social protection for health and climate risks, and disseminating best practices within and among countries are crucial, supported by robust evaluations to enhance progress.

Key Messages

- Current evidence highlights that climate change and related hazards have a significant impact on government, health insurance and household health-related expenditures.
- There is potential for greater promotive co-financing, through global aid, allocating health budgets to climate goals, expanding benefit packages to cover climate-sensitive conditions and adapting provider payment mechanisms to promote climate conscious behaviour. Climate finance can also be earmarked for health.
- Efforts towards integrative health and climate co-financing through adaptive social protection and regional contingency funds should be explored.
- Increased cross-sectoral collaboration, additional funding and skills for climate integration in health sector plans and budgets, and mainstreaming health within climate adaptation and mitigation plans, together with measuring co-benefits are needed to support co-financing.

## Introduction

The COVID-19 pandemic highlighted the universal vulnerabilities of health systems and current health financing arrangements, with reduced fiscal space for health (ILO, 2021) coupled with increased demands on health care services. COVID-19 also impacted food security and livelihoods, with implications for financial access to health care (Angeles-Agdeppa *et al*., 2022). Climate change presents similar risks to health systems and financing (Ebi *et al*., 2021). This requires the need for additional resources to address health conditions exacerbated by climate change (Ebi, 2008), and to build resilience, to protect supply chains, health workers and health care infrastructure to support continued service delivery (ILO, 2019; Codjoe *et al*., 2020) and to ensure continued access to services among the population.

Health financing can contribute to the achievement of climate goals through investments and incentives, which support climate mitigation and adaptation efforts. It is therefore necessary to reconfigure health financing arrangements, so that they can better protect health systems and households from the effects of climate change (Sheehan and Fox, 2020) and to support sustainability goals. However, at present, there is a considerable resource gap for climate adaptation and mitigation in the health sector (Alcayna and O’Donnell, 2022; Watkiss and Ebi, 2022). Climate finance—the funds and financing arrangements that support activities, programmes or projects to foster climate mitigation and adaptation globally—can also support health sector resource needs. The need for more synergistic strategies for climate and health financing has been recognized, in a newly adopted Declaration on Climate and Health and a set of Guiding Principles for Financing Climate and Health Solutions (COP28UAE, 2023a). Also, as a result of COP28, a number of international philantropies, governments and multilateral banks pledged a total of $1Bn to address the joint ‘climate and health crisis’ (COP28UAE, 2023b). Leveraging health and climate co-benefits, beneficial outcomes not directly related to the initial investments in climate and health, (Berrang-Ford *et al*., 2021), can best be achieved by moving away from isolated financing approaches and adopting intersectoral or co-financing strategies (Ebi *et al*., 2021). Cross-sectoral investment in joint goals can also promote efficiency (McGuire *et al*., 2019). Co-financing can encompass various financing functions, such as revenue generation, pooling of resources and purchasing strategies, to reduce fragmentation in financing and strengthen health care systems (Lie *et al*., 2015). However, it remains unclear what opportunities exist for co-financing, across which financing functions, and at which health system scales.

We define financing as the institutions and mechanisms that ensure financial flows towards populations, activities and programmes. We draw on the McGuire *et al*. (2019) concept of co-financing, namely financing arrangements in one sector designed to improve the outcomes of a different sector. This can be achieved through two co-financing approaches: ‘Promotive’, which uses funds from one sector to support the goals of another sector, addressing factors that affect the outcomes of both sectors; and ‘Integrative’, which involves joint or pooled funding from multiple budget holders to improve outcomes in two or more sectors (McGuire *et al*., 2019).

Furthermore, we propose that co-financing can be either ‘strategic’ or ‘passive’. Co-financing is strategic when one sector proactively supports the goals of another by taking pre-emptive measures to reduce risks and enhance resilience. In contrast, it is passive when one sector’s funding indirectly supports the goals of another, e.g. by addressing the consequences of climate-related hazards after they occur, or through inaction.

As with McGuire *et al*. (2019), we explore the potential for co-financing through revenue collection (leveraging climate resources for health gain), pooling (combining funds from different sectors) and/or purchasing functions (expanding benefits packages and/or implementing climate-adjusted payment mechanisms).

While a recent review identified a range of co-financing arrangements for health from other sectors, none covered climate goals or climate financing for health goals (McGuire *et al*., 2019). In this paper, we seek to identify the range of co-financing arrangements that have been used to support climate and health goals based on a rigorous review (Hagen-Zanker and Mallett, 2013) using a modified version of the McGuire co-financing framework. We also summarize the available evidence on enablers and barriers to implementation to guide future health and climate co-financing.

## Methods

### Approach for the study

This study used a rigorous, evidence-focused approach, similar to Hagen-Zanker and Mallett (2013). This approach aims to combine some of the core features of systematic review (including structured searches, clear inclusion criteria and transparency), while allowing for greater flexibility and reflexivity.

The literature review set out to identify co-financing arrangements for health and climate goals as per the above framework. Specifically, we identified three broad co-financing arrangements with five strategic/passive applications, which guided our review, as set out in Table 1.

**Table 1.** Overview of broad areas of co-financing guiding the review adapted from (McGuire *et al*., 2019)

Co-financing approaches	Strategic/Passive
1. Promotive: health financing for climate-related goals within the health sector	1.1 Strategic—health financing is budgeted and allocated towards risk reduction through mitigation or adaptation, and/or purchasing arrangements are re-designed to incentivize climate conscious behaviour 1.2 Passive—health financing supports climate goals through inaction, or funding the consequences of climate change or hazards *ex post*, or contributing to climate change through carbon emissions
2. Promotive: climate finance for health-related goals	2.1 Strategic—climate financing is budgeted and allocated towards health sector goals or towards climate goals within the health sector 2.2 Passive—climate financing supports health goals through co-benefits which are not built into program goals
3. Integrative: health and climate financing for joint goals through revenue collection, pooling or purchasing	3.1 Strategic—cross-sectoral collaboration relating to one or more of the financing functions to support joint goals

### Search strategy

The first step in the search strategy was identifying key terms across the three co-financing arrangements. The key terms for climate-related goals pertain to the nature of climate change and climate hazards. Terms also reflect the goals of adaptation, mitigation and resilience, and were guided by a previous systematic review of carbon reduction strategies for the health sector (Islam, 2020). The key terms for health system goals were informed by the authors’ knowledge of these areas and recent reviews (Islam, 2020; Singh *et al*., 2021). The key terms were initially piloted and refined to avoid ambiguous terms which could identify irrelevant studies (e.g. ecological). The final set of terms can be found in Table A1 in the Appendix.

**Figure 1.**
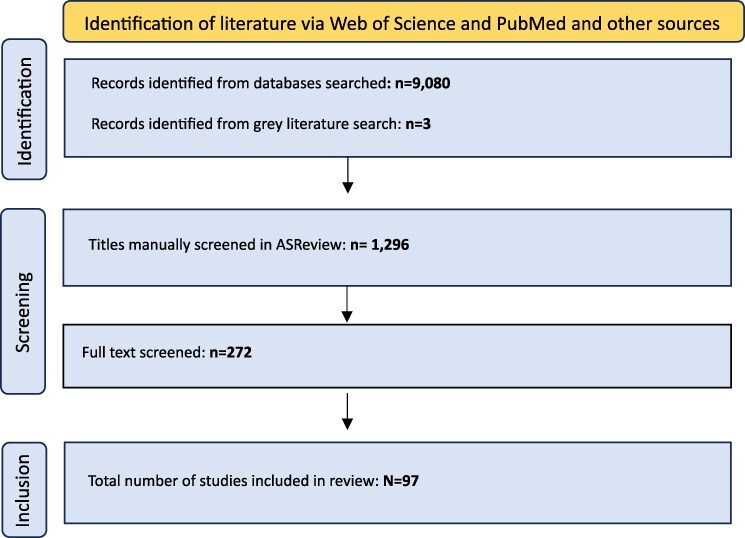
Flowchart of the systematic narrative review process

Combined searches were run in May and June 2023 for each of the three co-financing approaches in Web of Science and PubMed for the past 10 years covering international literature from all countries globally. For the three co-financing approaches, described in Table 1, we combined (1) search terms 1 and 3; (2) search terms 2 and 4; and (3) search terms 2 and 3 and (1 or 4) (Table A1). ASReview, an Artificial Intelligence-powered systematic review tool, was used to assist the screening of the literature. ASReview uses the information provided by the reviewer on the relevance of an article to order unscreened articles in terms of relevance. For each co-financing approach, the combined search results (a total of 9083 titles) were uploaded to ASReview and 1296 records were manually screened in ASReview (Figure 1).

In addition to the searches in PubMed and Web of Science, we identified references from the reference list of included articles, and grey literature was also searched from the websites of the following climate funds: Green Climate Fund, Least Developed Country Fund, Special Climate Fund, Global Environmental Facility Trust Fund Strategic Priority for Adaptation, World Bank managed Trust Fund, The Adaptation Fund, International Monetary Fund, Climate and Health Fund and European Union Solidarity Fund. The websites of United Nation agencies, including the World Health Organisation, United Nations Development Programme, United Nations Population Fund, United Nations Childrens Fund, World Food Programme and United Nations Environment Programme (UNEP), and other organizations, including the World Bank, Asian Development Bank, African Development Bank were also reviewed. Google searches were conducted using the keywords ‘climate co-financing’, ‘health and climate (co) financing’ and ‘health and climate finance’.

As a final step, a full-text screening of 272 papers was conducted.

We included papers that described one or more of the co-financing arrangements included in Table 1. We excluded articles that estimated the cost of responding to climate change or climate hazards, such as cost of illness studies, or studies estimating the economic impact for a single health facility without focusing on a particular financing mechanism. While we reviewed the international literature, we were interested in drawing lessons for the most climate vulnerable low- and middle-income countries (LMICs). We extracted information regarding the details of the co-financing arrangement, including the geographic location (countries, regions), country income group and geographic scale of the arrangement, the sources of funding, the climate and/or health goals targeted and, where available, enablers and barriers to implementation. The final screening resulted in the inclusion of *N* = 97 articles.

## Results

### Overview of the literature

Most examples were from LMICs (*n* = 56), followed by high-income countries (*n* = 27) and global examples (*n* = 13) (Supplementary file). The majority of examples were from Asia (*n* = 21, including 7 from China), Africa (*n* = 12) and the Americas (*n* = 15, including 9 from North America). Only a few studies from Europe (*n* = 3) were included.

In terms of the sources of health financing and types of climate finance, studies focused on carbon credits and pricing (*n* = 19), social protection schemes (*n* = 15), government funding (*n* = 10), national health insurance (*n* = 7), household out-of-pocket payments (OOPs, *n* = 6) and household private health insurance contributions (*n* = 6). Only a few studies focused on international aid (*n* = 4) and multiple funding sources (*n* = 3).

We included a total of 36 studies on health financing for climate goals (promotive), out of which most studies focused on air pollution (*n* = 12) or climate change in general (*n* = 14). A few studies looked into extreme weather events, such as droughts (*n* = 1), floods (*n* = 1) or hurricanes (*n* = 4), with two studies looking at a variety of climate hazards.

Most of these articles reported on passive co-financing arrangements (health financing supporting climate goals through inaction, or funding the consequences of climate change or hazards ex post) (*n* = 19). A number of studies (*n* = 10) described strategic co-financing (health financing budgeted and allocated towards risk reduction, and/or purchasing arrangements that are redesigned to incentivize climate conscious behaviour) to build resilience or sustainability within the health system or at the household level. One study described hypothetical health financing measures to build climate resilience.

We included a total of 39 studies on climate financing for health goals (promotive), out of which most examples looked into clean cooking and the use of improved cookstoves (*n* = 7). Some studies (*n* = 5) further focused on addressing the mental health effects of extreme weather events, access to water, sanitation, hygiene (WASH) (*n* = 5) and access to health care or health insurance (n = 6).

Our evidence review found that most co-financing arrangements for health and climate were promotive: climate or health sector funds were used to support the goals of the other sector. There were only three studies reporting integrative models of co-financing.

### Promotive: health financing for climate goals

#### Strategic

There was limited evidence of health financing being used to build health system sustainability and/or resilience to climate change. Our review identified examples of international aid and domestic health financing supporting climate adaptation within the health system. We also identified a number of adaptations made to health financing arrangements to account for climate change (Table 2).

**Table 2.** Overview of co-financing for health and climate identified through the review

Co-financing arrangements	Strategic/Passive	Enablers	Barriers
1. Promotive: Health financing for climate-related goals within the health sector	1.1 Strategic		
	Global aid		Allocation of climate-related health aid is not aligned with climate vulnerability Difficulty coordinating across multiple agencies Gaps in the skills to assess climate impacts and plan for adaptation Resource constraints/ restrictions on use of funds.
	Financing for climate adaptation at the subnational health system level Exempting disaster victims from insurance premiums Expanding benefit package to include climate sensitive conditions Disinvesting in fossil fuels within the health sector Adapting provider payment to incentivize climate conscious behaviour	Adequate levels of funding earmarked for climate goals Staff with climate knowledge Funding flexible and responsive to needs Creating climate and health institutions to facilitate joint planning	
	1.2 Passive Government, health insurance or household health expenditures linked to the consequences of climate change, waivers and exemptions from user charges/insurance premia for those impacted by climate hazards	Centrally managed schemes to ensure consistency and portability of benefits (exemptions/waivers)	Poor areas in most need not always able to compete for funding Wealthier households more likely to access insurance—inequity and impact of OOP worse for poor Confusion about entitlements when eligibility rules differ across administrative areas Delays in the waiver approval process Short-term nature of disaster response mechanisms, despite enduring health consequences Portability of health financing for those displaced by climate change
2. Promotive: Climate finance for health-related goals	2.1 Strategic Global aid and airline tax Carbon tax, fossil fuel subsidy removal earmarked for health	Earmarking of revenues for health can enhance social acceptability (tax)	Limited number of accredited health institutions can apply for funding (aid) Barriers for small domestic applicants (aid) Lack of engagement between health and climate stakeholders (all) Lack of awareness of health within multilateral climate funds (aid) Volatility in carbon markets may limit long-term health financing (tax) Green taxes will reduce in the long term as emissions reduce (tax)
	2.2 Passive Carbon credits and offsets invested with health co-benefits Cash transfers for mitigation with health co-benefits Social protection schemes improving health/health insurance coverage Health effects of climate insurance	Careful project implementation (carbon credits) Large-scale projects may be more profitable (carbon credits) Adequate monitoring of health impacts (all) Broader measures of carbon (carbon credits) Ensuring community buy-in (carbon credits) Clear statement of health goals (all) Budgeting for health monitoring and evaluation (all)	Vulnerable groups face barriers to access (insurance) Claims process can worsen mental health (insurance)
3. Integrative: Health and climate financing for joint goals through revenue collection, pooling or purchasing	3.1 Strategic		
	Carbon and health tax Adaptive social protection and health insurance Regional climate and health emergencies fund	Effective communication of health-benefits of social protection Streamlining data systems (insurance)	Complexity and political economy challenges of taxing/pricing multiple externalities (tax) Insufficient understanding of health-related benefits among social protection beneficiaries Dependencies between climate and pandemic risks impacting fund sustainability (regional fund). Challenge quantifying pandemic risk and risk dependencies (regional fund)

##### Global financing

An estimated 7% of bilateral health Official Development Assistance (ODA) is targeted at climate adaptation (Beyeler and Guinto, 2021). However, due to misreporting, this may be less in real terms. Less than 1% of bilateral health aid was targeted at climate mitigation (Beyeler and Guinto, 2021). Only 2.84% of the aid targeting the 20 most climate vulnerable countries was aimed at the health effects of climate change (Gupta *et al*., 2017). Due to the nature of aid reporting, analyses of aid disbursements rely on donor reports of intentionality regarding the funds, rather than an assessment of how the funds are actually used on the ground. Therefore, it is difficult to know how these funds were invested in practice.

##### Domestic health financing

We found only two examples of domestic health financing being used to support climate adaptation or mitigation within the health sector. The most widely documented example was the US Centre for Disease Control and Prevention (CDC) BRACE (Building Resilience Against Climate Effects) initiative to support capacity for climate adaptation in 18 public health jurisdictions. States with BRACE funding were found to be further along with climate and health programming than those without (Vo *et al*., 2022). Based on this experience, a number of barriers and enablers to implementation were identified. Enablers included adequate levels of earmarked funding for climate adaptation activities (Mallen *et al*., 2022). This was to avoid funds being reassigned to other priorities. As health department staff had limited time, funding for dedicated staff to work on climate and health activities was also found to be important. Flexibility of funding to be responsive to changing circumstances (Mallen *et al*., 2022), together with being ready to exploit opportunities for additional local-level funding (for natural resources, energy, land use and transport, which are often better funded) for health was also reported.

However, a number of challenges to implementation were also noted, including difficulties coordinating across multiple agencies; gaps in the skills required to assess climate impacts on public health and plan for adaptation; restrictions on the use of funding; complex fiscal and contracting procedures; and protracted hiring timelines, all of which constrained the use of funds (Mallen *et al*., 2022). Equity concerns were also raised as BRACE funding was not awarded to rural health departments, as they were less able to compete for funding. This was due to lower baseline performance and population size, both of which were criteria for funding. They were also less able to recruit and retain staff (Vo *et al*., 2022). Errett *et al*. (2022) reported that the states and territorial health authorities with a climate and health plan did not have more funding than those without, concluding that states may have insufficient resources to implement plans. However, sometimes climate hazards can provide an impetus for investing in climate resilience, with the heat and wildfires in California serving to support the approval of the 2022 budget proposal for funding surveillance of climate sensitive conditions and for local health departments and communities to develop climate and health resilience plans (Vo *et al*., 2022).

We found only one example from a low- and middle-income setting. The Government of Bangladesh has established a national Climate Change Trust Fund, which funds health system resilience building among other projects nationally, managed by a Climate Change and Health Promotion Unit within the Ministry of Health (WHO, 2018). This involves earmarked government health sector funding for climate goals. The nature of investments and experience with this financing arrangement are, however, to date, undocumented.

##### Adaptations to health financing arrangements

We found some evidence of health financing arrangements being adapted to promote sustainability and/or future climate resilience, in terms of modifications to revenue collection, population coverage and purchasing.

In Egypt, to enable the expansion of coverage of financial protection schemes during times of disaster, those impacted by disasters are eligible for exemptions within the Egypt Universal Health Insurance Project (Bank, 2020). The universal health insurance scheme is part-funded through earmarked taxes including from tobacco, toll roads and car licencing fees. The eligibility criteria were determined in the absence of a climate hazard (ex ante).

South Africa is currently considering expansion of the benefit package of the national health insurance scheme to include climate-sensitive conditions and mental health (Dos Santos *et al*., 2022). However, these authors also highlighted the lack of capacity and time within the government to tackle climate change in the health sector as well as resource constraints for making these adjustments.

At the household level, climate change can push households towards enrolling in private health insurance schemes as a means of offering future protection against health care expenditures associated with climate hazards. Evidence from China shows a significant increase in the demand for private health insurance associated with air pollution (Chang *et al*., 2018; Zhao and Xue, 2020; Wang *et al*., 2021; Jia and Yan, 2022). The effects are higher for wealthier and female-headed households (Wang *et al*., 2021) and for larger households in urban areas (Zhao and Xue, 2020). While this strategy enhances the resilience of insured households to the economic consequences related to pollution-induced ill-health, it does nothing to promote sustainability. Furthermore, there is a concern about the equity effects of such strategies, as wealthier households are more likely to obtain coverage, with those most in need falling short of financial protection.

The purchasing function of health financing provides a lever through which to incentivize mitigation and adaptation strategies within the health sector. Existing examples relate mostly to procurement among global agencies, and are focused on reducing carbon emissions. For example, the UN established a Sustainable Procurement in the Health Sector initiative, which encourages the inclusion of environmental criteria in health product and service procurement across the UN (UNDP, 2020). Country-level initiatives funded by Swedish SIDA in Guatemala, Moldova, Tanzania, Vietnam and Zambia aim to reduce greenhouse gas emissions in the supply chain among others. The UK’s National Health Service (NHS), to achieve the target of net zero by 2040, is reviewing provider payment mechanisms to understand the opportunities to drive environmental change through incentives (NHS, 2022). Similarly, Egypt has introduced a green health insurance system strategy informed by the national Go Greener Initiative, which includes criteria on energy efficiency, waste, telemedicine, renewable energy and use of recyclables as part of the accreditation and enrolment standards for the universal health insurance scheme (Bank, 2020). Tax rebates and economic incentives were highlighted as a means to promote climate conscious behaviour at the individual and institutional levels in South Africa (Dos Santos *et al*., 2022).

Fossil fuel disinvestment is also being considered within the health sector as a way of increasing sustainability, in the UK’s NHS (NHS, 2022). In Germany, private health insurance fund reserves were not invested in coal but guidelines did not preclude investments in oil or natural gas (Schneider *et al*., 2021). While such initiatives can contribute to the sustainability of the health sector, there is a need for monitoring and transparency regarding investments (Schneider *et al*., 2021). Investing in prevention and primary care has also been identified as an effective way of reducing emissions in the health sector (Rasheed *et al*., 2021).

#### Passive

Most of the literature reported on how health financing was used or adapted to meet the needs arising from climate change or climate hazards ex-post. We found evidence of health financing addressing the consequences of climate change by default (through inaction), or as part of the disaster response in the wake of a climate hazard at the national and household levels (Table 2).

##### National level (government and insurance funding)

There is evidence of increased levels of public spending (government and/or national health insurance) on health, to meet the additional demand for care among those affected by climate change in a number of countries. For example, there was a rise in national health insurance claims associated with air pollution in Korea (Kim *et al*., 2023) and Taiwan (Liu and Ao, 2021), and with floods (Yoshida *et al*., 2023) and earthquakes (Hasegawa *et al*., 2019) in Japan. In the USA, air pollution was associated with higher per capita levels of Medicare and Medicaid expenditure (Apergis *et al*., 2018), and hurricanes resulted in a substantial increase in public health expenditures in affected counties for up to a decade afterwards (Deryugina, 2017). In lower-income settings, a study of 15 Economic Community of West African States (ECOWAS) countries found that carbon emissions increased public health care expenditure though no effect was detected on private health care expenditure (Alimi *et al*., 2020). Another study across developing countries reported a positive association between environmental pollution and total health expenditure (Ampon-Wireko *et al*., 2022).

To protect victims of climate hazards, adjustments to health financing arrangements have also been made in the aftermath of a disaster. An example comes from Japan following the Great East earthquake, when the national government funded a 2-year exemption from co-payments, with local government contributions (Matsuyama *et al*., 2018). The scheme initially covered all impacted persons and then focused on lower-income groups. The government introduced a similar scheme for victims of the 2018 flooding (Yoshida *et al*., 2023). In the USA, a state-by-state emergency Medicaid waivers scheme was implemented offering 5 months cover to hurricane Katrina evacuees (Quast and Mortensen, 2012). The effects of these schemes were generally positive, with increased utilization and greater financial protection for beneficiaries. However, challenges were noted, including different eligibility rules across states in the USA resulting in confusion (Quast and Mortensen, 2012), eligibility criteria limiting access to affected populations including childless adults, delays in the waiver approval process, and a reliance on funding from hurricane-impacted states rather than federal support (Park, 2005). Centrally managed financing mechanisms may ensure consistency of benefits across geographies and avoid inequities as compared to local ones (Quast and Mortensen, 2012). A common concern is the short-term nature of disaster response mechanisms, despite the enduring health consequences of climate hazards (Gan *et al*., 2021).

##### Household level (Out of Pocket Payments (OOP))

There is also evidence of increased OOPs associated with climate change at the household level. A study across 49 African countries reported that a 1% increase in the level of greenhouse (CO_2_) emissions could increase OOP health expenditures by 0.423% (Ezeruigbo and Ezeoha, 2023). The same study also noted that higher-income countries, those with lower climate risk and lower age dependency, may experience a reduction in OOP. Increased household health expenditures were noted for pollution-associated illness in China, with a 1% increase in air pollution associated with a 10% increase in household health expenditure (Chen and Chen, 2021), and greater OOP was associated with the rise in climate-sensitive conditions in Bangladesh (Haque *et al*., 2013). The costs of medical and funeral costs associated with climate hazards could be substantial, amounting to an average USD 2190 in Indonesia (Dartanto, 2022). Displaced populations were found to be at particular risk of incurring OOP, when falling outside the cover of financial protection schemes. This was seen in relation to Medicare in the USA, which offers cover within specific geographic territories. Beneficiaries were reported to incur costs to access care when displaced outside of these areas due to a hurricane (Mellgard *et al*., 2019), despite the existence of the waiver scheme outlined above.

Studies also reported on the livelihood effects of climate change which were found to reduce household capacity to pay for health services, through shifts to lower-income activities (Jalal *et al*., 2021), reduced crop production and increased food prices (Dartanto, 2022), with rural agricultural households being most at risk, and earthquakes having the greatest income effects, followed by droughts and wildfires (Dartanto, 2022). Livelihood effects can result in out-migration of men for alternative employment, increasing income vulnerability of women (Rosen *et al*., 2021) and their ability to pay for and access health services.

### Promotive co-financing: climate finance that supports health goals

#### Strategic

Climate finance (including aid, social protection, carbon credits and carbon taxes) can be a potential source of funds for climate mitigation and adaptation in the health sector or for broader health sector investments (Table 2).

##### Global financing mechanisms

There is a limited evidence regarding multilateral and climate-related bilateral funding for health-related activities. An estimated USD 1431 million (4.9%) of multilateral and bilateral international adaptation finance was earmarked for health-related activities in a decade of climate adaptation financing (2009–2019) (Alcayna *et al*., 2023) in contrast to sectors like energy, transport, and agriculture that received more. Health-related climate investments comprised mostly grant funding (Beyeler and Schäferhoff, 2023). It was also found that those countries with greater climate vulnerability received relatively less funding (Alcayna and O’Donnell, 2022).

One of the constraints to accessing international climate funds within the health sector is the limited number of accredited health institutions which can apply for climate funding (Beyeler and Schäferhoff, 2023) as well as, in general, barriers for small domestic applicants to compete with international accredited institutions (Osuna, 2022). A lack of engagement between health and climate stakeholders and silos between respective Ministries also mean that countries often fail to include health within their National Adaptation Plans and Nationally Determined Contributions, which are pledges made by countries in the context of the Paris Agreement, detailing what they will do to help keep global heating under 1.5°C and adapt to the impacts of climate change (UNDP, 2023).

A lack of awareness of health within the multilateral climate funds has also been identified as a limiting factor, with an identified need for greater health advocacy within the climate community (Beyeler and Schäferhoff, 2023).

##### Carbon pricing

Carbon pricing includes carbon taxes and Emissions Trading Schemes (where companies have to acquire permits to emit greenhouse gases), as well as fossil fuel taxes or subsidy removal. Carbon pricing can generate substantial revenues which can, in theory, be used to fund a range of public health interventions as well as potentially increase health budgets. For example, Switzerland employed the funds generated through carbon taxes to alleviate the burden of healthcare insurance premiums for its citizens (Bank, 2023). Similarly, removing fossil fuel subsidies can free up fiscal resources for health care (Lie *et al*., 2015), generating potential health benefits (Ambasta and Buonocore, 2018). Other interventions that have been proposed in the literature include active transportation initiatives (Coomes *et al*., 2022), subsidization of healthy food categories (Springmann *et al*., 2018), water and sanitation infrastructure or energy poverty initiatives (Winkler, 2017). Carbon pricing yearly revenues currently exceed USD 95 billion. Revenue collection, however, is dominated by high-income countries, led by the EU Emissions Trading Scheme, with an annual collection of USD 42 billion. Among middle-income countries, Mexico’s carbon tax, for example, collected around USD 240 million in 2023.

There is a small number of studies comparing potential revenues from carbon pricing in a range of countries with funding needs related to the SDGs (Franks *et al*., 2018). Multi-country estimates are generally based on modelling exercises, however, and do not incorporate context-specific challenges or fully address considerations of global equity and climate justice as well as tax acceptability. Evidence suggests that earmarking carbon taxes for health can increase the social acceptability of carbon pricing in some contexts (Carattini *et al*., 2018). However, volatility in carbon markets can also be an important drawback when it comes to securing long-term financing for health interventions (Hodge and Clasen, 2016). Likewise, the long-term erosion of the base for green taxes as emissions are reduced can also pose challenges for reliance on carbon pricing revenues to finance growing health budgets (Speck, 2017).

#### Passive

The use of climate finance to fund initiatives outside of the health sector can result in health co-benefits (Table 2).

##### Cash transfers and climate insurance

Cash transfers, within social protection schemes, can be used to incentivize climate mitigation strategies at the household level, e.g. installing solar panels (Australia, China, India, Japan and the United Kingdom of Great Britain and Northern Ireland), or adaptation (e.g. heat proofing houses), with health co-benefits (Pega *et al*., 2015). Wider social protection schemes can also reduce climate-related migration by enhancing livelihoods and local labour market opportunities, which can alleviate the burden on health systems of increased demand due to migration (Silchenko and Murray, 2023).

Cash transfers targeting climate risks can be used to reduce the health consequences of climate hazards, by providing access to health care for displaced populations (Silchenko and Murray, 2023), or encouraging care uptake after a hazard (Macours *et al*., 2012; Langendorf *et al*., 2014). For example, during a drought in Kenya, cash transfers were given to orphans and vulnerable children to encourage health care attendance (Pega *et al*., 2015). One of the ways cash transfers can increase uptake of health care is through additional enrolment in health insurance schemes. For example, a study in Ethiopia found that participation in the social safety net programme increased enrolment in community-based health insurance by 16.3 percentage points among female-headed households in food-insecure and drought-prone rural areas (Mussa *et al*., 2022).

Insurance mechanisms introduced as part of climate adaptation can also affect public health. There is some (limited) evidence that index-based weather insurance can improve food security of farmers (Aheeyar *et al*., 2019). However, vulnerable groups including women experienced barriers to access related to illiteracy and gender norms (Lascano Galarza, 2020). In the context of flood insurance, the claims process itself has been identified as an aggravator of mental health illness post-flood (Foudi *et al*., 2017; McKenzie *et al*., 2022). More broadly, over-reliance on insurance mechanisms in the context of climate finance, especially private insurance, has been criticized as a distraction from real solutions (such as broad social safety nets) (Richards and Schalatek, 2018; Huber and Murray, 2023), serving private interests in higher-income countries in a context where climate risks are increasingly ‘uninsurable’. There is an opportunity here for learning from long-standing critiques of insurance-based approaches to health, which are based to an extent on common underlying concerns and failures.

##### Carbon credits

Carbon credits and related markets are a widely used, and highly controversial strategy to generate incentives for emission reduction, and have also been advocated as a strategy for generating resources to support environmental innovation and social goals.

The carbon credit market, although hard to assess in terms of value, generated around USD 10 billion in upstream investment in 2020 and is expected to grow considerably (Bank, 2023).

There is a growing body of evidence on the use of carbon credit sales to fund or incentivize emission reduction initiatives with health co-benefits, such as cookstove replacement (Aung *et al*., 2018), household water filters (Pickering *et al*., 2017) and community fire management. In addition to generating new sources of funds, it has been argued that this type of climate finance can have some advantages, such as supporting of longer-term and larger-scale project implementation (Freeman and Zerriffi, 2014; Hodge and Clasen, 2016) with integrated monitoring and evaluation which can potentially encourage more consistent implementation (Barstow *et al*., 2016; Newcombe *et al*., 2016).

However, there are only few examples where the health impacts of interventions funded via carbon credits or carbon pricing revenues have been rigorously evaluated. One study that reported on a randomized, carbon-financed cookstove intervention found no significant health impacts (Aung *et al*., 2018), potentially due to the incomplete adoption and a potential ‘rebound’ effect (or ‘Jevons paradox’), whereby more efficient cookstoves result in increased use.

There are a number of potential barriers to effective use of this type of climate finance for health promotion. Firstly, the use of results-based payment schemes which narrowly rely on quantifiable carbon emission reductions (tonnes of CO_2_ equivalents) can result in incentives that are misaligned with broader wellbeing outcomes for local communities and more specifically with health outcomes (Freeman and Zerriffi, 2014; Pickering *et al*., 2017). This can result, for example, in clean cookstove projects not distributing the type of cookstoves that would be most effective at reducing indoor air pollution. Equally, carbon credit-funded household water treatment could potentially lead to households switching from water boiling towards a less safe treatment in order to obtain carbon credit income, leading to negative health impacts (Hodge and Clasen, 2014). Careful project implementation can avoid the latter type of negative impacts by restricting eligibility to households that were not previously carrying out any water treatment, although it involves a trade-off in terms of mitigation effectiveness.

Secondly, the use of climate finance instruments and funding streams might also require relatively large-scale projects to ensure profitability. This can conflict with health goals in some cases where small, context-adapted interventions are most appropriate (Freeman and Zerriffi, 2012). The deployment of larger-scale interventions can also create challenges for the involvement of local actors in project delivery.

There are also challenges related to the monitoring and evaluation of health impacts. Although many carbon crediting projects provide integrated monitoring and evaluation, health outcomes monitoring can be particularly complex, and meeting the appropriate standards set by health bodies can require trained staff and additional investment. Projects that do not budget specifically for health monitoring risk inadequate monitoring and underdelivering on health-related outcomes (Hodge and Clasen, 2016; Aung *et al*., 2018).

Enablers to effective use of climate credits for health include the use of broader and more context-relevant measures of carbon which can, in theory, improve alignment, including for example, black carbon (a component of fine particulate matter or PM2.5 resulting from incomplete combustion of fossil fuels and biomass and which is a significant air pollutant) (Freeman and Zerriffi, 2014). The use of more holistic criteria for fund allocation as well as community buy-in into the project can also support improved alignment with health and social outcomes. However, these strategies risk increasing monitoring costs and complexity and reducing transparency. A clear statement of health goals prior to implementation and separate budgeting for health monitoring and evaluation can also support more synergistic projects (Hodge and Clasen, 2016; Ramanathan *et al*., 2017). Other strategies, such as acknowledgement of ‘suppressed demand’ effects (e.g. increases in demand that take place when barriers to consumption are removed, such as, for example, when income increases) (Hodge and Clasen, 2016) which can trigger an increase in demand for health-related goods and services as a result of improved livelihoods, helping to fund health interventions in low-income settings, although at some cost in terms of mitigation effectiveness.

However, it is importnat to be mindful that there is controversy regarding the effectiveness of carbon credits for climate change mitigation, their volatility and lack of transparency, as well as the power imbalances and processes of dispossession that can be driven by carbon projects and markets (Dunlap and Sullivan, 2020; Fleischman *et al*., 2021; Anguelovski and Corbera, 2023).

### Integrative health and climate co-financing

We found few empirical examples of integrative health and climate co-financing.

Hypothetical studies of a combined carbon and health tax (a tax on food products in proportion to their CO_2_ footprint and a summary health score) demonstrated the potential to generate health and climate co-benefits (Wang *et al*., 2015; Faccioli *et al*., 2022). However, challenges included the complexity of appropriately taxing/pricing multiple externalities with cross-sectoral interconnections, and political economy challenges of doing so (Sterner *et al*., 2019).

The Ghana’s Livelihood Empowerment Against Poverty (LEAP) 1000 cash transfer programme involving bimonthly payments to poor households with vulnerable children, orphans or pregnant women was paired with the National Health Insurance Scheme (NHIS), offering a waiver of all NHIS fees for card processing, premiums and renewals for LEAP beneficiaries (Tia *et al*., 2019). This led to increased health insurance enrolment among adults by 15 percentage points, including among the lowest socio-economic groups. However, challenges included insufficient understanding of NHIS benefits among beneficiaries and providers, meaning that sometimes LEAP beneficiaries were charged for services. The study recommended better streamlining of data systems and renewal timelines for both LEAP and NHIS as well as improved program communication.

At the regional level, the EU Solidarity fund was established to fill fiscal gaps in responding to floods and earthquakes and was extended to public health emergencies following COVID-19 (Hochrainer-Stigler *et al*., 2023). Countries received funds in proportion to the public funding used to respond to the disaster. The fund disbursed 530 million euros in response to COVID-19. However, a number of challenges were identified. First, there are dependencies between climate risks and COVID-19: those receiving payouts from the fund were more susceptible to the effects of future climate hazards, due to the large fiscal stimulus packages they had implemented during COVID-19. Second, COVID-19 substantially depleted the EU Solidarity fund, reducing the availability of funds for future climate hazards. To be effective, dependencies between health and climate risks need to be quantified and the fund capitalization increased accordingly. However, the quantification of risk in the case of pandemics is inherently more difficult than for natural disasters.

## Discussion

This is the first review of co-financing arrangements for health and climate goals. We proposed an adapted version of the McGuire framework to classify arrangements into promotive or integrative, and passive or strategic. This framework proved useful in classifying the evidence and mapping out co-financing strategies for health and climate reported in the literature, together with their potential challenges and enablers. Despite the substantial effects of climate change on health and the potential for action in the health sector to reduce climate risk (Kivioja *et al*., 2023), the evidence was primarily focused on documenting passive co-financing arrangements, including the health financing consequences of inaction. We found more limited examples of strategic co-financing to reduce risk and build resilience and sustainability within the health system, and only three integrative co-financing examples which require coordination and collaboration between sectors. The evidence also suggests that the levels of funding being channelled through strategic co-financing arrangements are very limited: with only a small fraction of international aid, and small levels of national funding supporting climate action in the health sector. The focus on documenting the health financing consequences of inaction is consistent with that found in the wider literature where emphasis is on assessing the impacts of climate change on health-related outcomes, and of climate inaction, rather than on evaluating mitigation and adaptation strategies. The limited number of co-financing studies may also reflect the general difficulties of inter-sectoral financing outlined elsewhere (McGuire *et al*., 2019).

Our review aimed to identify enablers and barriers to implementation. However, much of the literature described co-financing mechanisms without a critical appraisal of their implementation. There was also more frequent identification of barriers, and challenges of implementation, than there was of enablers.

Taken as a whole, however, a number of important lessons have emerged from this review that need consideration in the design of strategic co-financing mechanisms [be they promotive (health to climate; or climate to health) or integrative] to ensure they effectively meet health sector needs in relation to climate mitigation/adaptation, and overcome identified barriers. These include institutional/governance issues, such as involving staff with climate and health sector knowledge in the design and implementation of co-financing arrangements, and the alignment and/or linkage of information systems across sectors (particularly for integrative co-financing). While joint institutions/platforms were seen to enable cross-sectoral collaboration for co-financing, this has to be balanced against the potential additional administrative costs of new administrative structures.

A cross-cutting concern across co-financing mechanisms, is how to ensure funds are targeted to areas of greatest need and that groups most impacted by climate change can benefit. Clear communication about entitlements emerged as critical enabler in this respect, together with ensuring the consistency of entitlements across subnational administrative units.

Flexibility in funding, both in terms of what the funding can be used for but also where it can be used, given population displacement and mobility in the wake of climate hazards, was also found to be important. Rigid budgets and lack of financial autonomy have also been identified as barriers to co-financing in other sectors (McGuire *et al*., 2019). Our study found that long-term adjustments to financing arrangements, rather than short-term emergency response, will be needed to address ongoing health needs following a climate hazard.

Regarding the use of climate finance for health goals, it will be important to overcome barriers to access from institutions/domestic applicants, and vulnerable groups at the local level, by expanding accreditation of health institutions, and building capacity at national and local levels to mainstream health into climate change adaptation plans (Watkiss and Ebi, 2022). To maximize the benefits for health in terms of revenue and public health impact, careful monitoring and reporting of co-benefits will be key to avoid misalignment between health, and climate mitigation and adaptation goals. When relying on climate finance to support health goals, it is also important for the health systems community to keep in mind long-term trends affecting climate funding availability, including potential volatility in carbon markets and the erosion of revenues associated with successful emission reduction, in order to adequately plan for complementary sources of funding (Speck, 2017). While there is potential for adaptive social protection schemes, inculding safety nets, cash transfers and insurance schemes which are targeted to climate risks, to integrate health-related benefits, this needs to be weighed against the risk of prematurely burdening new and developing programmes with multiple objectives (Ulrichs *et al*., 2019).

While the regulatory environment was reported to be an issue affecting co-financing in other sectors (McGuire *et al*., 2019), this did not emerge as relevant in this study, likely because most of the studies were focused on promotive rather than integrative co-financing, where there was no change to funding channels. Though co-financing is sometimes proposed as enabling efficiency gains (McGuire *et al*., 2019); in practice, we did not find any evidence on the efficiency of co-financing arrangements in our review, maybe due to the difficulty in estimating costs and outcomes across multiple sectors (Marsh *et al*., 2016). While one might expect greater efficiency gains from integrative approaches, the associated political economy and administrative challenges, together with the need for multi-hazard risk assessment, may make strategic promotive approaches more readily achievable in the short term.

Recent commitments of Global Environmental Facility funding for health sector mitigation and adaptation in a range of countries are encouraging developments towards a more strategic promotive co-financing. There is also the potential to consider blended financing as a mechanism to bring together international funding from climate finance initiatives and/or global health intiatives to service climate and health goals, while mobilizing funding from public development banks (Xu *et al.*, 2020). Blending grants and lending helps increase concessionnality and ease the terms of financing for countries that have little access to financial markets. There is also some early and limited evidence of crowding in of private funding through instruments such as social impact bonds (SIBs). SIBs are a mechanism by which to shift financial risk from service providers to investors, with investors underwriting service providers’ based on their ability to deliver on positive social outcomes (Fraser *et al.*, 2018).

At country level, there is also an opportunity to consider how current incentives and purchasing arrangements, including result-based financing mechanisms, can encourage climate conscious mitigation and adaptation behaviour when designing health financing strategies.

Our review has a number of limitations. Given the nature of the study, we included a diverse range of sources including grey literature and government reports. Although these sources were thoroughly assessed, this makes it more difficult to replicate the search strategy. Our definition and framework for co-financing, although drawing from an existing framework (McGuire *et al*., 2019), included passive co-financing, which may not typically be considered as co-financing. However, we consider it important to make the distinction between passive and strategic co-financing: ultimately through inaction the health sector is financing climate-related goals, but is not doing so in a way which is compliant with the Sendai framework, which supports the global response to climate change. This approach also potentially undermines the fiscal resilience of the sector. Financing of health goals and interventions through carbon credits can also be considered, arguably, a source of ‘passive’ co-financing, as carbon credit programmes are not explicitly designed to support health, and fund health interventions only based on their resulting emission reductions.

Most of the studies we identified were quantitative in nature, examining the effect of climate change or hazards on health financing arrangements or the impact of climate finance initiatives on health. There were much fewer qualitative or mixed methods pieces. Although we did not undertake a quality appraisal as part of this review, most studies assessing the linkages between air pollution or emissions and health care expenditure controlled for selected confounders, including individual and household controls.

Many of the LMIC experiences were extracted from programme documents describing plans, with no research yet evaluating their implementation. As a result, there was often little detail on the co-financing arrangements themselves, their impact, implementation or enablers and barriers, and only a few studies reported on the potential pathways to the reported outcomes. There is, therefore, an urgent need for more research documenting and evaluating co-financing experiences to guide policy. In particular, although there is emerging literature from LMIC settings, there is a need for more research in these settings and consideration of the specificities of co-financing in these settings, where climate change poses the greatest risk to health, despite these populations contributing the least to carbon emissions.

We used ASReview an AI-powered system in our review, and did not manually review all identified articles. ASReview sorts references based on relevance, following initial training by the reviewer. Once a certain number of irrelevant references are identified, the reviewer can decide to stop the review as remaining references are likely to be irrelevant. In this case, we stopped our review after screening 50 consecutive irrelevant references, hence we are fairly confident not to have missed references.

## Conclusion

Our study identified a range of potential co-financing approaches for health and climate goals in the literature and key enablers and barriers to their effective implementation. Co-financing is critical to filling the financing gap for climate mitigation and adaptation in the health sector (Neufeldt *et al*., 2021), and achieving recent COP28 funding pledges. Ultimately, however, there is no one best co-financing approach. In practice, a mix of options will be needed to meet COP28 financing commitments among the donor community and among LMICs, including global aid financing, adaptations to national health financing arrangements and leveraging multiple climate finance arrangements for health. Our review has highlighted some of the issues to consider in designing and implementing these schemes to maximize their benefit for health systems; and drawn attention to some of the limitations of specific arrangements, such as the volatility and long term sustainability of carbon pricing revenue. There is an urgent need for future research focused on evaluating co-financing strategies, in terms of the impact on health and climate goals. Implementation studies to understand how these mechanisms work in practice, and how context shapes this, together with political economy issues are also needed. More insights on the efficiency and cost of different co-financing approaches can help guide investments. Greater evidence on the economic and environmental benefits of investing in health (Watkiss and Ebi, 2022), and of the health co-benefits of climate action will also encourage greater co-financing (Beyeler and Schäferhoff, 2023).

## Supplementary Material

czae044_Supp

## Data Availability

No new data were generated or analysed in support of this research.

## Reflexivity statement

The author group includes six female and three male researchers. The authors differ widely in terms of their seniority within their respective institutions, research experience and thematic expertise. Author order has been established based on individual author contributions as opposed to seniority. All researchers are from high-income countries, although most of them work within international institutions with a strong focus on global health, development and health in LMICs. The inclusion of authors from LMICs would have been desirable although, given the global focus of the review, there was no particular area to target for author inclusion. Authors were included based on their involvement in earlier discussions and ongoing work as part of international conferences (the One Sustainable Health Conference in Lyon, 2023), International Working Group 5 on financing. We acknowledge the existence of biases in terms of participation and barriers to inclusion in such forums for research and policy discussion, which the authors are committed to fighting.

## Appendix

**Table A1.** Overview of key search terms by concepts

Concept	Key search terms			
**1a.Climate goals: mitigation and adaptation**	emissions adj2 (mitigat* or reduc* or abate* or curtail* or lower) carbon adj2 (mitigat* or reduc* or abate* or curtail* or lower) CO2 adj2 (mitigat* or reduc* or abate* or curtail* or lower) renewable ‘energy efficien*’ ‘fuel efficien*’ decarboni* ghg	‘sustainable procurement’ ‘sustainable design’ ‘sustainable transport’ ‘cool roof’ ‘green roof’ ‘green building’ ‘solar power’ ‘smart metre’ ‘smart meter’ ‘eco-design’ ‘greenhouse gas*’	insulat* ‘low carbon’ ‘green design’ bioenergy ‘climate preparedness’ ‘climate mitigation’ ‘climate adaptation’ ‘climate resilience’ ‘air condition*’ ‘sustainable supply chains’ ‘sustainable waste management’	‘tree canop*’ ‘vegetation’ ‘shade’ ‘environmental restoration’ ‘UV protect*’ ‘climate proof’ ‘white roof’ ‘bio fuel’ ‘sustainable water’ ‘environmental sustainability’
**1b. Climate goals: climate hazards**	‘natural hazard’ ‘natural disaster’ ‘extreme weather’ drought landslide storm	flood* ‘extreme heat’ ‘extreme temperature’ hurricane tsunami	wildfire tornado ‘heat wave’ ‘cold wave’ cyclone desertification	downpour earthquake pollution ocean acidification deforestation
**2. Climate financing**	‘heat incentive*’ ‘climate fund*’ ‘green bond*’ ‘(climate AND “social protection”)’ ‘green finance’ ‘green fiscal policy’ ‘disaster financ*’ ‘climate risk’	‘(climate AND “loan guarantee*”)’ ‘climate financ*’ ‘flood insurance’ ‘(climate AND microfinance)’ ‘adaptation fund*’	‘(climate AND “nutrition programme” AND cash)’ ‘(weather AND insurance)’ ‘drought insurance’ ‘climate insur*’	‘least developed country fund’ ‘special climate change fund’ ‘carbon credits’ ‘climate financ*’ ‘carbon tax*’ ‘fuel tax*’
**3. Health financing**	‘health financing’ ‘development assistance for health’	‘external financing’ ‘health aid’	‘health insurance’ ‘out-of-pocket payment’	‘out-of-pocket expenditure’ ‘universal health coverage’
**4. Health and health system goals**	health* morbidity mortality	disease* illness* ‘burden of disease*’	DALY* ‘life year*’	QALY* death*
	hospital clinic doctor general practitioner	‘health facility’ ‘health centre’ nurse	‘health care’ ‘health system’ ‘health service*’ patient	surgery surgical ‘emergency service’
