# Supplementary material for: Climate and health: a path to strategic co-financing?
Source: Health Policy Plan. 2024 Nov 18;39(Suppl 2):i4–i18. doi: 10.1093/heapol/czae044 (PMC11959176; doi:10.1093/heapol/czae044)
Supplement: czae044_Supp [file czae044_supp.zip › Health Policy and Planning - table [50].docx]

Health Policy and Planning – Responses to reviewers’ comments

| **Reviewer 1** | **Response** |
| --- | --- |
| Thank you for the opportunity to review this paper. There were aspects to the paper that I enjoyed but there are a few areas that I feel need revision. | Thank you for the feedback. We have done our best to address the comments raised. |
| **Methodology** |  |
| The paper adopts a “systematic narrative review” drawing on Wong et al 2013. But Wong et al are describing a meta narrative review, which is – I think – something different? It wouldn’t matter so much, but I’m not sure whether the aims of the paper and  the purpose behind a meta narrative review (and the steps described by Wong et al) are aligned as well as they could be. For example, the main aim of the paper seems to be to identify enablers and barriers to implementation of co-financing models in the context of health and climate change. This should be quite straightforward but a clear answer to the question is not forthcoming, possibly because the authors follow the Wong et al steps in the Discussion and focus on strengths/weaknesses and policy recommendations (NB: the key phrase in Wong et al “If appropriate, offer recommendations for policy and practice”). I’m not really convinced that policy recommendations are necessary – or at least not frame the key points identified as policy recommendation but, instead, go back to the main aim and frame them as enablers and barriers. I would also query why a ‘narrative’ review is chosen rather than a ‘systematic’ review (See Green, Johnson and Adams 2006 “Writing narrative literature reviews…’). So, I have a few ?? over the choice of methodology and how it meets the main aims of the paper. | Our review is a narrative review as it sought to bring together a variety of evidence across multiple disciplines at the nexus between health and climate financing. The approach used aims to combine some of the core features of systematic review (including structured searches, clear inclusion criteria and transparency) while allowing for greater flexibility and reflexivity.  We have removed reference to Wong, and adapted the search approach section of the paper to more clearly reflect the approach we used in this study. |
| **Methods** |  |
| A significant, if understated, element to this paper is the use of McGuire to create a matrix (I’m not sure model is the right word) with Promotion, Integration, Strategic and Integrative dimension.  This does help to synthesise the literature. However, I think the presentation of results could be simplified. The way the authors present the results – 1. Promotive…1.1 Strategic…Global aid…etc.. –  is not that clear. I also found it difficult remembering what promotive, integrative, etc meant and was continually referring back to the Introduction (Table 1 is ok, but even that is not as clear as it might be – maybe the terms promotive, integrative etc are just not intuitively meaningful?) | We have defined the terms in both Tables 1 and 2 as well as in the text. The headings in the text of the paper, also clarify what is meant by promotive either within the title or in the text that follows (see 1.1, 1.2, 2.1, 2.2). We are reluctant to adapt the terms promotive / integrative as they are derived from the McGuire framework which we draw upon. We have edited the first part of the results to remind the reader of definitions. We have also reformatted headings to try and improve clarity. |
| I was very interested to read that the authors used AI powered ASReview. I’ve not used this tool. It would be interesting to read any reflections on its use. to have a ? | We have added some reflection on the use of AS Review to the Discussion. AS Review is a useful tool to organise the literature. Using training information from reviewers as to which articles are relevant, it orders articles in order of relevance. Once a certain number of irrelevant articles have been reviewed the reviewer can determine that the remaining articles will be irrelevant, saving time in the review process. |
| As this is a literature review, I’m not sure why key informants were approached on an ad hoc basis or Pathfinder was used in order to identify case studies. This step of the method seems quite unsystematic and, I think, muddies the analysis (which should be about the literature). | Given that the topic of co-financing for climate and health is understudied, we reached out to key informants to try and identify relevant grey literature which we may have otherwise missed. However, no evidence was identified in this way. We have therefore removed this from the paper. |
| If case studies are important, how can the authors know that they identified all, or even the best ones? One example was the use of the BRACE example. I wasn’t clear if the authors were saying that they only found two examples of domestic health literature from the literature, or whether they were saying that there various efforts (literature review and interviews with informants and experts) had only yielded two examples (if the latter, then I think that’s a problem). | We only found two examples of domestic health financing being used to support climate goals in the literature review. As mentioned above, we did not include any case studies from other sources. |
| **Discussion** |  |
| It’s clear that much of the work has been focussed on the Results section and fitting the literature into the matrix/model. I feel that the Discussion section has not been given equal attention. Indeed, there are very few actual discussion points. I don’t think it’s useful here (pace Wong et al) to summarise the findings in the opening paragraph. | We feel it important to commence the Discussion by highlighting the key findings and contributions of the paper. However, we have now tried to do this more systematically and comprehensively in relation to each of the papers three objectives. Specifically, we have added a section summarising implementation issues that need to be considered, and links to the wider literature. |
| But that aside, paragraph two is quite weak. It starts with a confirmation that was likely never in doubt, nor the unequal impact. It introduces the Sendai framework without any explanation and mentions some international initiatives. And then we’re into the limitations section and the recommendations. To repeat an earlier point, I think this is where the Wong et al methodology is not that helpful for this paper. What we really want in this section is a return to the main aims and some clear answers to the RQs. We also want some reflections on the literature – why is there so much focus “on how health financing was used or adapted to meet the needs arising from climate change or climate hazards ex-post” for example? I can’t tell if the recommendations are taken from literature identified in the literature review? | We have now clarified the purpose of the review which was to adapt and apply a co-financing framework, map out the type of co-financing arrangements reported in the literature, and summarise reported barriers and enablers to implementation. We have now provided a summary of findings in relation to each in the discussion and have made reference to the wider literature, where relevant.  We have also added some reflections on why the focus has been on a passive approach to date, and in the concluding section, we highlight that a mix of approaches will be needed in practice. |
| As I mentioned, it may be possible to reframe the recommendations in terms of enablers and barriers? That would allow the authors to return to the earlier parts of the paper and the core aims. | We thank the reviewer for this suggestion. As advised, we have now added a column in Table 2 on enablers and barriers, and return to this in the discussion, and tie this to the recommendations made. |
|  |  |
| **Reviewer 2** | **Response** |
| This paper tackles the important question of how to leverage and optimize climate and health financing for synergistic impacts / co-benefits. It summarises the current evidence base on different co-financing models, provides a useful framework for categorising them, and distils some of the key lessons from what has been developed and, in some cases, implemented. As such, it is a valuable contribution to the field. Below are some comments and suggestions to strengthen the paper and the interpretation of results. | We thank the reviewer for their feedback. |
| **Major comments** |  |
| It is not clear from the analysis where there is most potential benefit from co-financing. The implicit assumption that it is always worth investing in strategic co-financing models would need to be nuanced as this is not necessarily the case (especially with the evidence of unintended negative consequences that the paper brings forward, and potentially high opportunity costs). | This is an important point. We feel that co-financing for health and climate will be essential to address arising health and health system risks from climate change. It is also critical to address resource gaps and meet recent COP28 pledges. We now state this in the Discussion. We also make the point there is no one best approach, and a mix of approaches will be needed depending on country context. |
| Rather, it would be useful to identify **what the current evidence base is suggesting may hold most value for strategic co-financing (given that passive co-financing will happen anyway).** In terms of policy implications, it seems possible that there may be immediate value in embedding health impact assessment and mitigation measures in climate related investments, rather than necessarily trying to design a fully integrative health and climate investment with demonstrable joint outcomes (with all the coordination and transaction costs that would imply). | We now make the point in the paper that there is no one best approach, and a mix of approaches will be needed depending on country context.  We agree with the reviewer that promotive financing is more readily achievable than fully integrative, and have added a sentence to this effect in the Discussion. |
| The climate sector’s areas of intervention and financing models would benefit from greater explanation and description, given the journal’s health audience. Several references to climate-related intiatives and financing models are made without explanation and make it more difficult to follow (e.g. carbon credits, carbon pricing, combined carbon and health taxes, Sendai framework, etc).  The ‘passive’ co-financing category is more of an indirect cost to the other sector (resulting from inaction or ineffective prevention) rather than an investment. The paper fully recognises this, but it strikes me that this may need rethinking if the framework is meant to shape more prospective investments to achieve health and climate goals. | We have now added explanations of climate finance initiatives in the text. Regarding the passive co-financing we agree with the reviewer and identify this in our limitations. However, for the reasons stated, we feel it important to document, as the health sector is still supporting climate goals through these mechanisms, albeit less effectively. |
| It is difficult to fully appraise and engage with the review without the summary tables that lists the included papers. These should be part of the paper, at least as supplementary files. | We have added a table with all included studies to the appendix of the paper. |
| The discussion is missing literature on outcome-based financing models (such as social impact bonds, etc) and what is relevant or different with these climate/health co-financing models that have been/are being implemented or proposed. Given the multiple climate and health financing instruments, joint or what some are calling ‘blended financing’ models among international funders also seem relevant (where different international financing sources with divergent goals blend resources, such as multi-lateral development bank loans and Global Fund or GAVI grant funds) to leverage multiple funding streams and optimise joint impact. | While the innovative financing mechanisms mentioned do hold potential for resource generation for health and climate, we did not find evidence of these being used to support co-financing. We have added a comment indicating the potential of these approaches and need for more evidence. |
| **Minor comments** |  |
| It is not always clear what the primary objective of the paper is, i.e. to document models of co-financing, develop a framework, and/or identfy enablers and barriers to implementation. It would be good to be more consistent in describing the objective and reflecting on the results in the discussion. | The paper proposes a framework for health and climate co-financing, and uses this to map out the evidence to see which co-financing approaches have been used and what the enablers and barriers to implementation were. We have now tried to make this clearer throughout the text, and better align the discussion to this. |
| The framing refers to the potential for co-financing to generate revenues and efficiency gains. Yet, the focus appears to be on the former, while I think there is more evidence of co-financing being a means for the latter. | We found no evidence on this in the review. We now make reference to this in the discussion, and identify this as an area for future research. |
| I would also suggest being clearer about distinguishing between empirical and hypothetical co-financing models throughout. One example on p.9 “removing fossil fuel subsidies can free up fiscal resources for healthcare”: are the retained studies suggesting it has freed up resources that were invested in healthcare or are they suggesting that they could? | Almost all studies are empirical, but we now identify more clearly where studies are hypothetical. |
| I would find it useful to see where we have more evidence of climate-health co-financing, considering health financing functions (revenue generation, pooling, purchasing) and climate investments (adaptation, mitigation). | We have added information in the table describing studies whether funding is for mitigation or adaptation. We did not include a column on financing functions, as this does not apply so readily to climate finance. |
| P. 7: the section on the studies reporting associations between carbon emissions/ pollution and health expenditure raises many questions about confounders. It would be helpful to address these and explain how they were dealt with. | We have added a comment on methods for the quantitative studies in the Discussion section. |
| P. 9: “Carbon pricing yearly revenues exceed USD 95 billion”: assume this is a global estimate? In which countries are these revenues predominantly generated? | We have added text detailing where the revenues are generated. |
| P.11: The inclusion of Ghana’s LEAP programme as an integrative co-financing model is very surprising. How is this climate-related? And if this social cash transfer programme is considered / included, I can think of many more such social protection interventions across the African region that would also meet the inclusion criteria (perhaps they are included in the long-list of studies). This raises some concerns about the search strategy and how broad the inclusion criteria are. | A number of studies have considered the effect of social protection schemes on health, but here there is an effort to link an ‘adaptive’ social protection scheme which was aimed at drought victims to a health insurance scheme. We did not find other studies linking adaptive social protection with health insurance schemes. |
| P. 14: “Support for alternative income sources to improve livelihoods (…) especially for women”: I assume the explicit mention of women here is linked to the specific studies that considered certain groups of women, but I would expect this to be relevant to a broader set of socially disadvantaged groups, including certain groups of women among them. I would encourage more specificity about what the evidence tells us about which groups are most disadvantaged and what intersecting factors influence their heightened risks. | We have removed mention of women here. |
| P.14 “… incentives for climate-conscious investments in health sector”: in settings with severe fiscal constraints and large unmet healthcare needs, it is uncertain this would be the best of use of health resources (i.e. the health-related oppportunity costs would likely be too high). This may be where climate financing may be a more relevant source, as it may have different opportunity costs / alternative investment options. It may be worth including a more nuanced interpretation of these investments in different country settings / income levels. | We have tried to reflect on this in the conclusion where we highlight the choice of approach/es will be context specific. |
|  |  |
