# Supplementary material for: Climate and health: a path to strategic co-financing?
Source: Health Policy Plan. 2024 Nov 18;39(Suppl 2):i4–i18. doi: 10.1093/heapol/czae044 (PMC11959176; doi:10.1093/heapol/czae044)
Supplement: czae044_Supp [file czae044_supp.zip › HPP - table of included studies.docx]

| **Authors** | **Title** | **Country/ Region** | **Type of mechanism** | **Type of financing** | **Adaptation/ mitigation** |
| --- | --- | --- | --- | --- | --- |
| Aheeyar et al., 2019 | Making index-based flood insurance socially inclusive in Bangladesh: challenges and options | Bangladesh | Social Protection, Insurance | Climate financing for health goals | Adaptation |
| Alimi et al., 2020 | Environmental quality and health expenditure in ECOWAS | West Africa | Government funding and household | Health financing for climate goals | Adaptation |
| Ampon-Wireko et al., 2022 | The relationship between healthcare expenditure, CO2 emissions and natural resources: evidence from developing countries | LMICs | National health expenditure | Health financing for climate goals | Adaptation |
| Apergis et al., 2018 | U.S. state-level carbon dioxide emissions: Does it affect health care expenditure? | United States | Medicare/medicaid - government tax funding | Health financing for climate goals | Adaptation |
| Aung et al., 2018 | Effect on blood pressure and eye health symptoms in a climate-financed randomized cookstove intervention study in rural India | India | Carbon credits | Climate financing for health goals | Mitigation |
| Barstow et al., 2016 | Process evaluation and assessment of use of a large scale water filter and cookstove program in Rwanda | Rwanda | Carbon credits | Climate financing for health goals | Mitigation |
| Beyeler and Guinto, 2021 | Closing the gap on health-related climate financing | Global | Co-financing | Climate financing for health goals | Adaptation and Mitigation |
| Biosca and Brown, 2015 | Boosting health insurance coverage in developing countries: do conditional cash transfer programmes matter in Mexico? | Mexico | Social Protection, Insurance | Climate financing for health goals | Adaptation |
| Cai et al., 2016 | Gearing carbon trading towards environmental co-benefits in China: Measurement model and policy implications | China | Carbon trading | Climate financing for health goals | Mitigation |
| Carter, 2015 | Micro levies for public global goods | Global | Micro-levies | Climate financing for health goals | Adaptation and Mitigation |
| Chang et al., 2018 | Something in the Air: Pollution and the Demand for Health Insurance | China | Household private health insurance | Health financing for climate goals | Adaptation |
| Chen and Chen, 2021 | Cost of economic growth: Air pollution and health expenditure | China | National health insurance and household | Health financing for climate goals | Adaptation |
| Coomes et al., 2022 | Assessment of the health benefits to children of a transportation climate policy in New York City | United States | Carbon pricing | Climate financing for health goals | Mitigation |
| Dartanto, 2022 | Natural disasters, mitigation and household welfare in Indonesia: Evidence from a large-scale longitudinal survey | Indonesia | Household | Health financing for climate goals | Adaptation |
| Deryugina, 2017 | The Fiscal Cost of Hurricanes: Disaster Aid versus Social Insurance | United States | Medicare/medicaid - government tax funding | Health financing for climate goals | Adaptation |
| Dos Santos et al., 2022 | Climate change and health within the South African context: A thematic content analysis study of climate change and health expert interviews | South Africa | National health insurance | Health financing for climate goals | Adaptation |
| Errett et al., 2022 | Climate Change Adaptation Activities and Needs in US State and Territorial Health Agencies | United States | Government funding | Health financing for climate goals | Adaptation |
| Evans et al., 2016 | Cash transfers and health: Evidence from Tanzania | Tanzania | Social Protection, Cash transfers | Climate financing for health goals | Adaptation |
| Ezeruigbo and Ezeoha, 2023 | Climate change and the burden of healthcare financing in African households | 49 African countries | Household | Health financing for climate goals | Adaptation |
| Foudi et al., 2017 | The effect of flooding on mental health: Lessons learned for building resilience | Bangladesh | Social Protection, insurance | Climate financing for health goals | Adaptation |
| Franks et al., 2018 | Mobilizing domestic resources for the Agenda 2030 via carbon pricing | Global | Carbon pricing | Climate financing for health goals | Mitigation |
| Fraundorfer, 2015 | Experiments in global democracy: The cases of UNITAID and the FAO Committee on World Food Security | Global | Global governance | Climate financing for health goals | Adaptation and Mitigation |
| Freeman, 2012 | Carbon credits for cookstoves: Trade-offs in climate and health benefits | LMICs | Carbon credits | Climate financing for health goals | Mitigation |
| Freeman and Zerriffi, 2014 | How You Count Carbon Matters: Implications of Differing Cookstove Carbon Credit Methodologies for Climate and Development Cobenefits | LMICs | Carbon credits | Climate financing for health goals | Mitigation |
| Gan et al., 2021 | A scoping review of climate-related disasters in China, Indonesia and Vietnam: Disasters, health impacts, vulnerable populations and adaptation measures | China, Indonesia, Vietnam | Household | Health financing for climate goals | Adaptation |
| Gupta et al., 2017 | Adapting global health aid in the face of climate change | Global | Aid | Health financing for climate goals | Adaptation |
| Guzman and Clapp, 2017 | Applying personal carbon trading: a proposed “Carbon, Health and Savings System' for British Columbia, Canada | Canada | Personal carbon trading | Climate financing for health goals | Mitigation |
| Haque et al., 2013 | Health coping strategies of the people vulnerable to climate change in a resource-poor rural setting in Bangladesh | Bangladesh | Household | Health financing for climate goals | Adaptation |
| Hasegawa et al., 2019 | Worsening Health Status among Evacuees: Analysis of Medical Expenditures after the 2011 Great East Japan Earthquake and Nuclear Disaster in Fukushima | Japan | National health insurance | Health financing for climate goals | Adaptation |
| Hodge and Clasen, 2014 | Carbon financing of household water treatment: background, operation and recommendations to improve potential for health gains | LMICs | Carbon credits | Climate financing for health goals | Mitigation |
| Hodge and Clasen, 2016 | A Critical Review of Carbon Credits for Household Water Treatment | LMICs | Carbon credits | Climate financing for health goals | Mitigation |
| Jakob et al., 2016 | Carbon Pricing Revenues Could Close Infrastructure Access Gaps | Global | Carbon pricing | Climate financing for health goals | Mitigation |
| Jalal et al., 2021 | Does climate change stimulate household vulnerability and income diversity? Evidence from southern coastal region of Bangladesh | Bangladesh | Household | Health financing for climate goals | Adaptation |
| Jeuland and Pattanayak, 2012 | Benefits and costs of improved cookstoves: assessing the implications of variability in health, forest and climate impacts | LMICs | Carbon credits | Climate financing for health goals | Mitigation |
| Jia and Yan, 2022 | Effects of haze pollution and institutional environment on demand for commercial health insurance | China | Household private health insurance | Health financing for climate goals | Adaptation |
| Karhunmaa, 2016 | Opening up storylines of co-benefits in voluntary carbon markets: An analysis of household energy technology projects in developing countries | LMICs | Carbon credits | Climate financing for health goals | Mitigation |
| Kilburn et al., 2016 | Effects of a Large-Scale Unconditional Cash Transfer Program on Mental Health Outcomes of Young People in Kenya | Kenya | Social Protection, Cash transfers | Climate financing for health goals | Adaptation |
| Kim et al., 2022 | Effect of Ambient Air Pollutants on the Medical Costs of Allergic Rhinitis in Seoul, Korea | South Korea | National health insurance | Health financing for climate goals | Adaptation |
| Langendorf et al., 2014 | Preventing acute malnutrition among young children in crises: a prospective intervention study in Niger | Niger | Social Protection, cash or food transfers | Climate financing for health goals | Adaptation |
| Lascano Galarza, 2020 | Resilience to food insecurity: Theory and empirical evidence from international food assistance in Malawi | Malawi | Social Protection, aid | Climate financing for health goals | Adaptation |
| Liu and Ao, 2021 | Effect of air pollution on health care expenditure: Evidence from respiratory diseases | Taiwan | National health insurance | Health financing for climate goals | Adaptation |
| Macours et al., 2012 | Cash Transfers, Behavioral Changes, and Cognitive Development in Early Childhood: Evidence from a Randomized Experiment | Nicaragua | Social Protection, Cash transfers | Climate financing for health goals | Adaptation |
| Mallen et al., 2022 | Overcoming Barriers to Successful Climate and Health Adaptation Practice: Notes from the Field | United States | Government funding | Health financing for climate goals | Adaptation |
| Matsuyama et al., 2018 | Copayment Exemption Policy and Healthcare Utilization after the Great East Japan Earthquake | Japan | National health insurance | Health financing for climate goals | Adaptation |
| McKenzie et al., 2022 | Insurance Issues as Secondary Stressors Following Flooding in Rural Australia—A Mixed Methods Study | Australia |  | Climate financing for health goals | Adaptation |
| Mellgard et al., 2019 | Hurricanes and healthcare: a case report on the influences of Hurricane Maria and managed Medicare in treating a Puerto Rican resident | Puerto Rico | Medicare/medicaid - government tax funding | Health financing for climate goals | Adaptation |
| Mulchandani et al., 2019 | Effect of Insurance-Related Factors on the Association between Flooding and Mental Health Outcomes | United Kingdom | Social Protection, insurance | Climate financing for health goals | Adaptation |
| Mussa et al., 2022 | Impact of conditional cash transfers on enrolment in community-based health insurance among female-headed households in south Gondar zone, Amhara region, Ethiopia | Ethiopia | Social Protection, Cash transfers | Climate financing for health goals | Adaptation |
| Newcombe et al., 2016 | Innovations in Payments for Health Benefits of Improved Cookstoves | LMICs | Carbon credits | Climate financing for health goals | Mitigation |
| NHS, 2022 | Delivering a net zero national health service | United Kingdom | Government funding | Health financing for climate goals | Mitigation |
| Park, 2005 | Failing to Deliver: Administration’s Medicaid Waiver Policy Excludes Many Katrina Survivors and Provides No Guarantee of Full Federal Financing | United States | Medicare/medicaid - government tax funding | Health financing for climate goals | Adaptation |
| Pega et al., 2015 | Climate change, cash transfers and health | Global | Social Protection, Cash transfers | Climate financing for health goals | Adaptation |
| Pickering et al., 2017 | Climate and Health Co-Benefits in Low-Income Countries: A Case Study of Carbon Financed Water Filters in Kenya and a Call for Independent Monitoring | Kenya | Carbon Credits | Climate financing for health goals | Mitigation |
| Quast and Mortensen, 2012 | Emergency department utilization in the Texas Medicaid emergency waiver following Hurricane Katrina | United States | Medicare/medicaid - government tax funding | Health financing for climate goals | Adaptation |
| Raifman et al., 2021 | Mortality Implications of Increased Active Mobility for a Proposed Regional Transportation Emission Cap-and-Invest Program | United States | Carbon pricing | Climate financing for health goals | Mitigation |
| Ramanathan et al., 2017 | Wireless sensors linked to climate financing for globally affordable clean cooking | India | Carbon credits | Climate financing for health goals | Mitigation |
| Robinson et al., 2016 | Indigenous benefits and carbon offset schemes: An Australian case study | Australia | Carbon credits | Climate financing for health goals | Mitigation |
| Rosen et al., 2021 | "Burnt by the scorching sun": climate-induced livelihood transformations, reproductive health, and fertility trajectories in drought-affected communities of Zambia | Zambia | Household -OOP/livelihoods | Health financing for climate goals | Adaptation |
| Schneider et al., 2020 | Do private German health insurers invest their capital reserves of €353 billion according to environmental, social and governance criteria? | Germany | Private health insurance | Health financing for climate goals | Mitigation |
| Silchenko and Murray, 2023 | Migration and climate change – The role of social protection | LMICs | Social Protection | Climate financing for health goals | Adaptation |
| Speck, 2017 | Environmental tax reform and the potential implications of tax base erosions in the context of emission reduction targets and demographic change | HICs | Carbon pricing | Climate financing for health goals | Mitigation |
| Springmann et al., 2017 | Mitigation potential and global health impacts from emissions pricing of food commodities | Global | Carbon pricing | Climate financing for health goals | Mitigation |
| Ulrichs et al., 2019 | Building resilience to climate risks through social protection: from individualised models to systemic transformation | Ethiopia, Uganda, Kenya | Social protection | Climate financing for health goals | Adaptation |
| UNDP, 2020 | Sustainable Health Procurement Guidance Note | Global | Aid | Health financing for climate goals | Mitigation |
| Vo et al., 2022 | Addressing Capacity Constraints of Rural Local Health Departments to Support Climate Change Adaptation: Action Is Needed Now | United States | Government funding | Health financing for climate goals | Adaptation |
| Wang et al., 2021 | Energy Consumption and Health Insurance Premiums in China's Provinces: Evidence From Asymmetric Panel Causality Test | China | Household private health insurance | Health financing for climate goals | Adaptation |
| Wang et al., 2021 | Who is more important, parents or children? Economic and environmental factors and health insurance purchase | China | Household private health insurance | Health financing for climate goals | Adaptation |
| Wang et al., 2021 | Effects of SO(2) Pollution on Household Insurance Purchasing in China: A Cross-Sectional Study | China | Household private health insurance | Health financing for climate goals | Adaptation |
| WHO, 2013 | UN Initiative on Greening Procurement in the Health Sector: From Products to Services | Global | Aid | Health financing for climate goals | Mitigation |
| WHO, 2018 | Bangladesh National Health Adaptation Plan | Bangladesh | Government funding | Health financing for climate goals | Adaptation |
| Winkler, 2017 | Reducing energy poverty through carbon tax revenues in South Africa | South Africa | Carbon credits | Climate financing for health goals | Mitigation |
| World Bank, 2020 | Supporting Egypt’s Universal Health Insurance System | Egypt | National health insurance | Health financing for climate goals | Adaptation |
| Yoshida et al., 2023 | Effects of 2018 Japan floods on healthcare costs and service utilization in Japan: a retrospective cohort study | Japan | National health insurance | Health financing for climate goals | Adaptation |
| Yoshino et al., 2023 | Experiences of conditional and unconditional cash transfers intended for improving health outcomes and health service use: a qualitative evidence synthesis | Global | Social Protection, Cash transfers | Climate financing for health goals | Adaptation |
| Zhao, 2020 | Effect of air pollution on household insurance purchases. Evidence from China household finance survey data | China | Household private health insurance | Health financing for climate goals | Adaptation |
